# Biguanides are associated with decreased early mortality and risk of acute kidney injury in hospitalised patients with COVID-19: a nationwide retrospective cohort study in Japan

**DOI:** 10.1007/s10157-025-02755-z

**Published:** 2025-08-27

**Authors:** Mari Sugimoto, Hiroaki Kikuchi, Eisei Sohara, Koji Mizutani, Kavee Limbutara, Akihiro Hirakawa, Takayasu Mori, Koichiro Susa, Shuichiro Oya, Takefumi Suzuki, Shotaro Naito, Soichiro Iimori, Tatemitsu Rai, Kiyohide Fushimi, Shinichi Uchida

**Affiliations:** 1 Department of Periodontology, Institutes of Science Tokyo, Tokyo, Japan; 2 Department of Nephrology, Institutes of Science Tokyo, Tokyo, Japan; 3 Division of Nephrology and Renal Replacement Therapy, Department of Internal Medicine, Faculty of Medicine Vajira Hospital, Navamindradhiraj University, Bangkok, Thailand; 4 Department of Clinical Biostatistics, Institutes of Science Tokyo, Tokyo, Japan; 5 Department of Gastrointestinal Surgery, Graduate School of Medicine, The University of Tokyo, Tokyo, Japan; 6 Department of Nephrology and Hypertension, Dokkyo Medical University, Tochigi, Japan; 7 Department of Health Policy and Informatics, Institutes of Science Tokyo, Tokyo, Japan

**Keywords:** COVID-19, Biguanides, Mortality, Acute kidney injury

## Abstract

**Background:**

The most prescribed oral glucose-lowering medication worldwide is biguanide (BG), which shows potential for further therapeutic applications. The coronavirus disease 2019 (COVID-19) pandemic is a global public health emergency. Nevertheless, low-cost treatments against COVID-19 have not been established, with varying morbidity and mortality rates in each country.

**Methods:**

From the inpatient databases in Japan from September 2021 to March 2023, which includes the era following the development of COVID-19 vaccines, we extracted data from 168,370 patients with COVID-19 aged 20– < 80 years with diabetes mellitus treated with oral antidiabetic agents. The primary and secondary outcomes were 100-day in-hospital mortality and incidence of acute kidney injury (AKI) during hospitalisation, respectively. We compared outcomes in patients who received BG with those who did not using a logistic regression analysis and Cox proportional hazards under both propensity score-unmatched and matched cohorts.

**Results:**

The incidence of in-hospital death was significantly lower in the BG group (1.18%) compared with the non-BG group (2.41%) (*P* < 0.001). Similarly, the incidence of AKI during hospitalisation was significantly lower in the BG group (0.66%) compared to the non-BG group (1.12%) (*P* < 0.001). Kaplan–Meier analysis from the propensity score-matched cohort showed a significantly better survival rate in the BG group (adjusted HR, 0.619; 95% CI, 0.545–0.702; *P* < 0.001).

**Conclusion:**

In patients with COVID-19, oral biguanide use may be associated with reduced in-hospital mortality and AKI risk.

**Supplementary Information:**

The online version contains supplementary material available at 10.1007/s10157-025-02755-z.

## Introduction

The 2019-nCoV, a novel coronavirus, was initially identified in China and has since spread globally [1]. COVID-19 is caused by severe acute respiratory syndrome coronavirus 2 (SARS-CoV-2), a virus belonging to the *Betacoronavirus* genus [2]. The global challenge posed by COVID-19 is significant due to its complex transmission and absence of proven treatment. Whilst the large-scale production of vaccines is a significant achievement, the global distribution of vaccines [3] and virus mutations [4] continue to pose challenges in eradicating SARS-CoV-2. The overall case–fatality ratio stands at 1.38% (95% confidence interval [Cl], 1.23–1.53); however, the absolute number of deaths remains high due to the virus’s high transmission rate and increased risk of mortality amongst older individuals with comorbidities [5]. Although treatments such as remdesivir and dexamethasone are available or in development for severe COVID-19, early interventions are urgently required to prevent disease progression and long-term complications.

Acute kidney injury (AKI) frequently occurs in hospitalised COVID-19 patients [6]. It affects more than one-fifth of COVID-19 hospitalisations and is twice as common in patients requiring intensive care, often leading to approximately 10% requiring dialysis and high mortality rates [7].

Recent theoretical evidence suggests that metformin, an oral biguanide, may have various beneficial effects, including anti-viral [8], immune response [9], anti-cancer [10] and anti-hypertensive properties [11]. Metformin was discovered in 1922 [12] and introduced in the USA in 1995, becoming the most widely prescribed oral medication for reducing blood glucose globally [12]. Several clinical evidence have assessed the impact of metformin on COVID-19 patients, although opinions have varied [13, 14]. This discrepancy may be due to the complex interplay of factors influencing the prognosis of COVID-19, including medical facility infrastructure, healthcare personnel resources, and patient backgrounds [15] [15]. Therefore, obtaining a comprehensive national overview of the impact of COVID-19 on the population is crucial for identifying factors associated with mortality and improving COVID-19 management. The Diagnosis Procedure Combination (DPC) is a patient classification method developed in Japan to categorise inpatients in the acute phase of illness within the country’s healthcare system and facilitate evaluation and quality improvement [17].

We conducted a retrospective analysis of the impact of in-hospital biguanide use on COVID-19 patients, utilising a large nationwide patient cohort of 168,370 hospitalised individuals. To gain insights into biguanide treatment in COVID-19, we assessed the discharge/100-day in-hospital death and the incidence of AKI as the primary and secondary endpoints, respectively.

## Materials and methods

### Study design and settings

This retrospective observational cohort study used data from the nationwide administrative claims and the DPC database. In brief, > 1000 hospitals, including all 81 academic hospitals in Japan, contribute to the DPC database. Approximately 7,000,000 cases are added to the database annually and include almost 50% of all hospital admissions in Japan [17]. The DPC database contains the demographics, unique hospital identifier, diagnoses, outcomes, medications used, procedures performed, healthcare costs and several disease-specific data [18]. Moreover, diagnoses include the main diagnosis on admission, pre-existing comorbidities and postadmission complications, which are recorded separately with the International Classification of Diseases-10th (ICD-10) revision codes and text data in Japanese.

### Study participants

The patients were eligible for inclusion if they met the following criteria: [1] admitted to the hospitals participating in the DPC system from September 2021 to March 2023, [2] aged 20–80 years old, [3] had COVID-19 infection identified by the ICD-10 revision codes (version 2013) of B-342 and [4] were treated with oral antidiabetic agents, including biguanides, dipeptidyl peptidase-4 (DPP-4) inhibitors, sodium–glucose cotransporter type 2 (SGLT2) inhibitors, sulfonylureas (SU), alpha-glucosidase inhibitors (aGI), glucagon-like peptide-1 (GLP-1) analogues and thiazolidinediones (TZD).

**Fig. 1:**
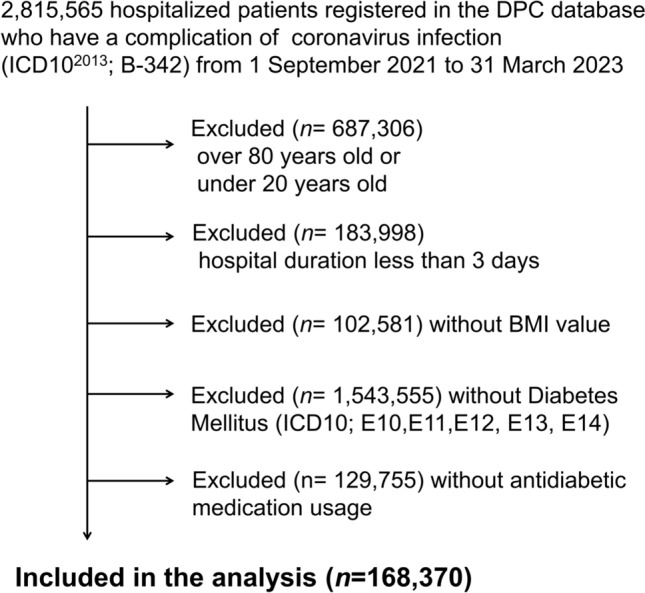
Flow chart of recruitment of participants and subjects studied. *DPC*, Diagnosis Procedure Combination; *ICD*, International Classification of Diseases; *BMI*, body mass index

We excluded patients who reached the outcome within 2 days and included 168,370 subjects (Fig. 1). All patient data were followed up until they reached the outcome or until they were discharged from hospitals within 100 days after admission. We only used data from their first admission for subsequent analyses for patients with multiple admissions at the same hospitals during the study period.

### Data collection and processing

Based on pathophysiological importance, data on demographics, existing comorbidities and several hospital characteristics were extracted for each patient. From the ICD-10 revision code (version 2013), existing comorbidities included hypertension, chronic kidney disease (CKD), coronary artery disease (CAD), cerebral infarction (CeI) and malignancies. We also extracted age, sex, body mass index (BMI) and Charlson score from the DPC system. The Charlson Comorbidity Index (CCI) is a method used to predict mortality by categorising comorbid conditions in a patient. It assigns a weighted score to various conditions based on their severity and impact on patient outcomes. In the context of the Diagnosis Procedure Combination (DPC) system, the Charlson score is adapted to assess patient severity and guide resource allocation. We also included medications, including BG, DPP-4 inhibitors, SGLT2 inhibitors, SU, aGI, GLP-1 analogues, TZD and salicylate, and insulin usage as hospital characteristics. All ICD-10 codes (Supplementary Table S1) and receipt numbers (Supplementary Table S2) for medication used in this study are summarised. Data acquisition is performed on August 30th, 2023.

### Study groups and study endpoint

We divided the patients into two groups based on the presence (*n* = 30,908) or absence (*n* = 137,642) of biguanide treatment during hospitalisation. The primary endpoint was in-hospital death occurrence within 100 days. Cases discharged alive within 100 days were considered non-fatal. The secondary outcome was AKI onset during hospitalisation.

Statistical methods used are demonstrated in Supplementary Information.

## Results

### Cohort characteristics

We selected 168,370 patients hospitalised with COVID-19 treated with antidiabetic medication during 2021–2023 (Fig. 1). The mean age of the cohort was 67.7 ± 10.6 years, and 68.2% (*n* = 114,894) were men, and 8.79% (*n* = 5,880) had CKD. The mean BMI was 24.8 ± 5.44 kg/m^2^. Table 1 shows the baseline characteristics of the patients between the two groups: Patients without biguanide (BG) treatment (No Biguanide) and Patients with biguanide treatment (Biguanide). Of the 168,370 patients, 30,908 (18.4%) received BG. Patients with BG treatment were younger, more likely to be women, had higher Charlson scores, higher BMI, higher prevalence of cerebral infarction (CeI), and lower prevalence of hypertension, CKD, cardiovascular disease, malignancies and pneumonia (Table 1). Patients with BG treatment were more likely to be treated with SU and TZD and less likely treated with DPP4 inhibitors, SGLT2 inhibitors and salicylate (Table 1). Amongst patients prescribed biguanides, approximately 75% received a daily dose between 250 mg and 1,000 mg. The mean daily dose was 785 mg, whilst the median was 750 mg (Supplementary Fig. 1). The proportion of insulin use was 56.3% in the group receiving biguanide treatment and 51.1% in the group without biguanide treatment (Table 1). The mean median BG dose during hospitalisation was 789 mg in insulin users and 780 mg in non-insulin users.

**Table 1:** Comparison of baseline population characteristics between groups

	All (*n* = 168,370)	No biguanide (*n* = 137,642)	Biguanide (*n* = 30,908)	
Variables				*P*
Patient demographics				
Age (SD)	67.7 (10.6)	68.1 (10.3)	65.9 (11.3)	< 0.001
Male sex, *n* (%)	114,894 (68.2)	94,447 (68.7)	20,447 (66.2)	< 0.001
BMI (kg/m^2^)	24.8 (5.44)	24.6 (5.50)	25.5 (5.13)	< 0.001
Smoking, n (%)	89,225 (53.0)	73,061 (53.1)	16,164 (52.3)	0.006
Charlson score	0.98 (1.45)	0.96 (1.48)	1.03 (1.33)	< 0.001
Comorbidities				
Hypertension, *n* (%)	67,159 (39.9)	55,390 (40.3)	11,760 (38.0)	< 0.001
Malignancy, *n* (%)	39,795 (23.6)	32,631 (23.7)	7164 (23.2)	0.037
CKD, *n* (%)	14,796 (8.79)	14,432 (10.5)	364 (1.18)	< 0.001
Cardiovascular Disease, *n* (%)	29,727 (17.7)	25,789 (18.8)	3938 (12.7)	< 0.001
Cerebral Infarction, *n* (%)	12,786 (7.59)	10,070 (7.33)	2716 (8.79)	< 0.001
Pneumonia, *n* (%)	7113 (4.22)	5918 (4.31)	1195 (3.87)	0.0005
COPD, *n* (%)	2460 (1.46)	2023 (1.47)	437 (1.41)	0.459
Other medications				
*DM meds*				
DPP4	123,992 (73.6)	104,893 (76.3)	19,099 (61.8)	< 0.001
SGLT2i	54,135 (32.2)	44,506 (32.4)	9629 (31.2)	< 0.001
SU	17,056 (10.1)	13,548 (9.9)	3508 (11.3)	< 0.001
aGI	24,035 (14.3)	20,466 (14.9)	3569 (11.5)	< 0.001
GLP1	4158 (2.47)	3414 (2.48)	744 (2.41)	0.45
TZD	2951 (1.75)	1347 (0.98)	1604 (5.19)	< 0.001
Salicylate	43,841 (26.0)	36,864 (26.8)	6977 (22.6)	< 0.001
Insulin, *n* (%)	87,615 (52.0)	70,216 (51.1)	17,399 (56.3)	< 0.001

Normally distributed variables are presented as mean ± standard deviation (SD). Categorical data are presented as counts and percentages. *BMI* body mass index, *CKD* chronic kidney disease, *COPD* chronic obstructive pulmonary disease, *DM* diabetes mellitus, *DPP4* dipeptidyl peptidase-4 inhibitors, *SGLT2i* sodium–glucose cotransporter type 2 inhibitors, *SU* sulfonylureas, *aGI* alpha-glucosidase inhibitors, *GLP-1* glucagon-like peptide-1 analogues *TZD* thiazolidinediones

### In-hospital BG use is associated with lower in-hospital mortality rates in admitted patients with COVID-19 under unmatched cohort

Amongst the 168,370 patients, in-hospital death occurred in 3,689 patients (2.19%) during the 100-day follow-up period. In the univariate, unadjusted analysis, patients receiving BG were less likely to experience the primary endpoint event compared with those who did not (odds ratio, 0.49; 95% CI, 0.44–0.54) (Table 2). Subsequently, we aimed to identify variables significantly associated with the primary outcome. All Pearson scores were < 0.5, indicating no strong correlation between any two variables in Table 1. In multivariate logistic regression analyses, BG use was significantly associated with lower mortality in all models (Table 2).

**Table 2:** Association of BG use and in-hospital death in the univariate and multivariable analyses in the overall study cohort

	Unadjusted OR	*P*	Model 1^a^		Model 2^b^	
			Adjusted	*P*	Adjusted	*P*
	(95% CI)		OR		OR	
			(95% CI)		(95% CI)	
Biguanide treatment,	0.49	< 0.001	0.60	< 0.001	0.56	p < 0.001
yes (ref = no)	(0.44, 0.54)		(0.540, 0.669)		(0.500, 0.640)	
Age			1.04	< 0.001	1.04	< 0.001
(per 1-year increase)			(1.035, 1.044)		(1.036, 1.046)	
Female			0.76	< 0.001	0.76	< 0.001
(ref = male)			(0.701, 0.823)		(0.691, 0.833)	
Smoking			1.13	0.004	1.07	0.110
(ref = no)			(1.056, 1.217)		(0.985, 1.161)	
BMI			0.94	< 0.001	0.96	< 0.001
(per increase of 1)			(0.928, 0.943)		(0.956, 0.974)	
Charlson score			1.34	< 0.001	1.21	< 0.001
(per 1-year increase)			(1.323, 1.364)		(1.190, 1.240)	
CKD,					1.45	< 0.001
yes (ref = no)					(1.289, 1.623)	
Chronic obstructive pulmonary disease,					1.12	0.359
Yes (ref = no)					(0.877, 1.401)	
Malignancy,					1.47	< 0.001
Yes (ref = no)					(1.349, 1.611)	
Pneumonia,					2.48	< 0.001
Yes (ref = no)					(2.194, 2.799)	
Salicylates treatment					0.98	0.648
Yes (ref = no)					(0.886, 1.078)	
SGLT2 treatment,					0.83	< 0.001
Yes (ref = no)					(0.757, 0.916)	
DPP4 treatment,					0.96	0.404
Yes (ref = no)					(0.867, 1.060)	
αGI treatment,					0.81	< 0.001
Yes (ref = no)					(0.727, 0.904)	

^a^Model 1 adjusted for age, sex, history of smoking, BMI and Charlson score

^b^Model 2 adjusted for all variables in model 1 plus comorbidities of hypertension, cardiovascular disease, chronic kidney disease (CKD), chronic obstructive pulmonary disease, malignancy, cerebral infarction (CeI), pneumonia and use of salicylates, vasopressor, SGLT2 inhibitor, DPP4 inhibitor, aGI treatment and insulin use

**Fig. 2:**
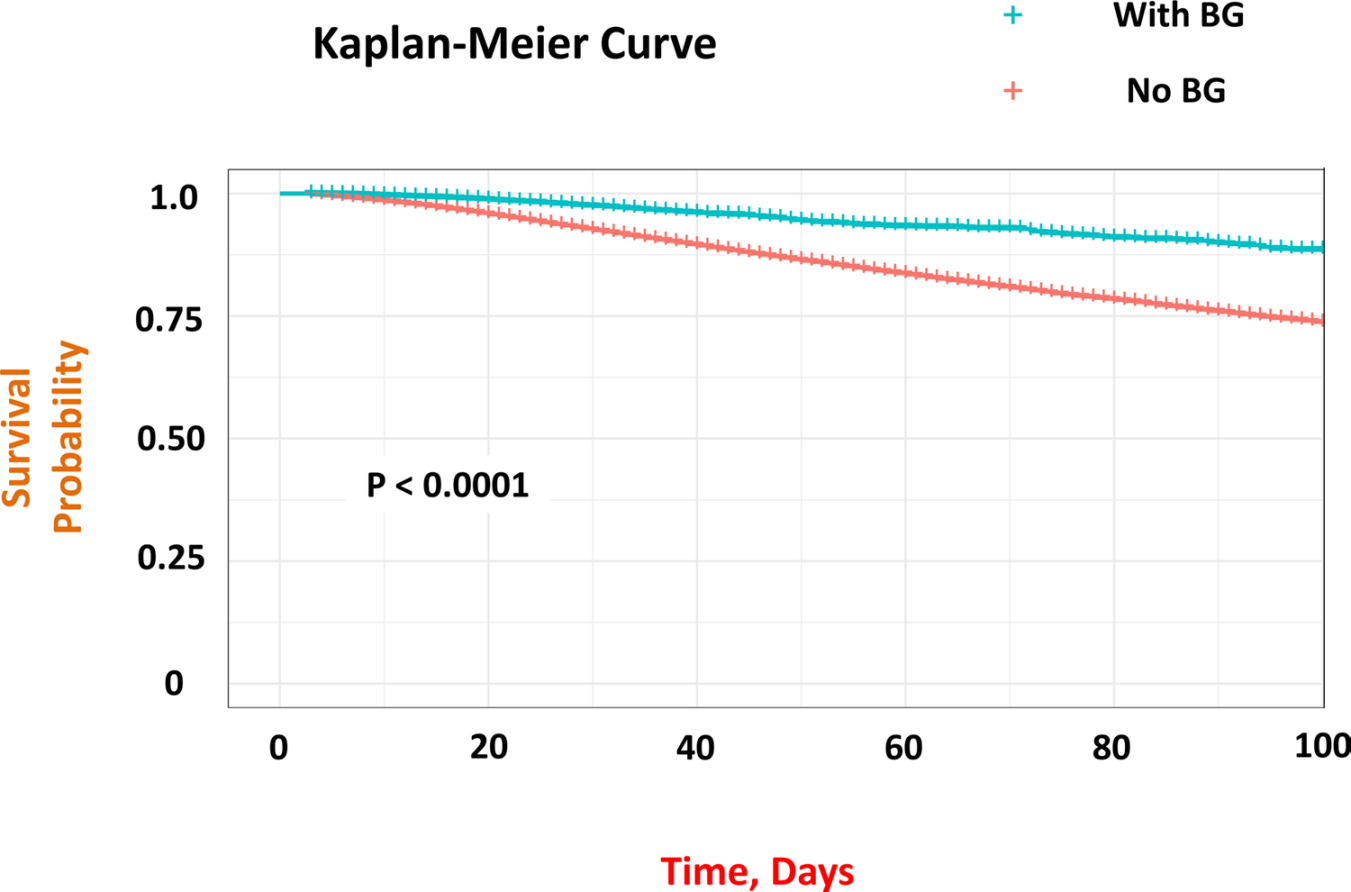
Kaplan–Meier estimates of all-cause mortality for admitted patients with COVID-19 with and without biguanide use under unmatched cohort. Patients with COVID-19 treated with BG had a statistically significant higher survival rate than those without BG treatment (*P* < 0.001, Log-rank Chi-square = 155) *BG* biguanide

**Fig. 3:**
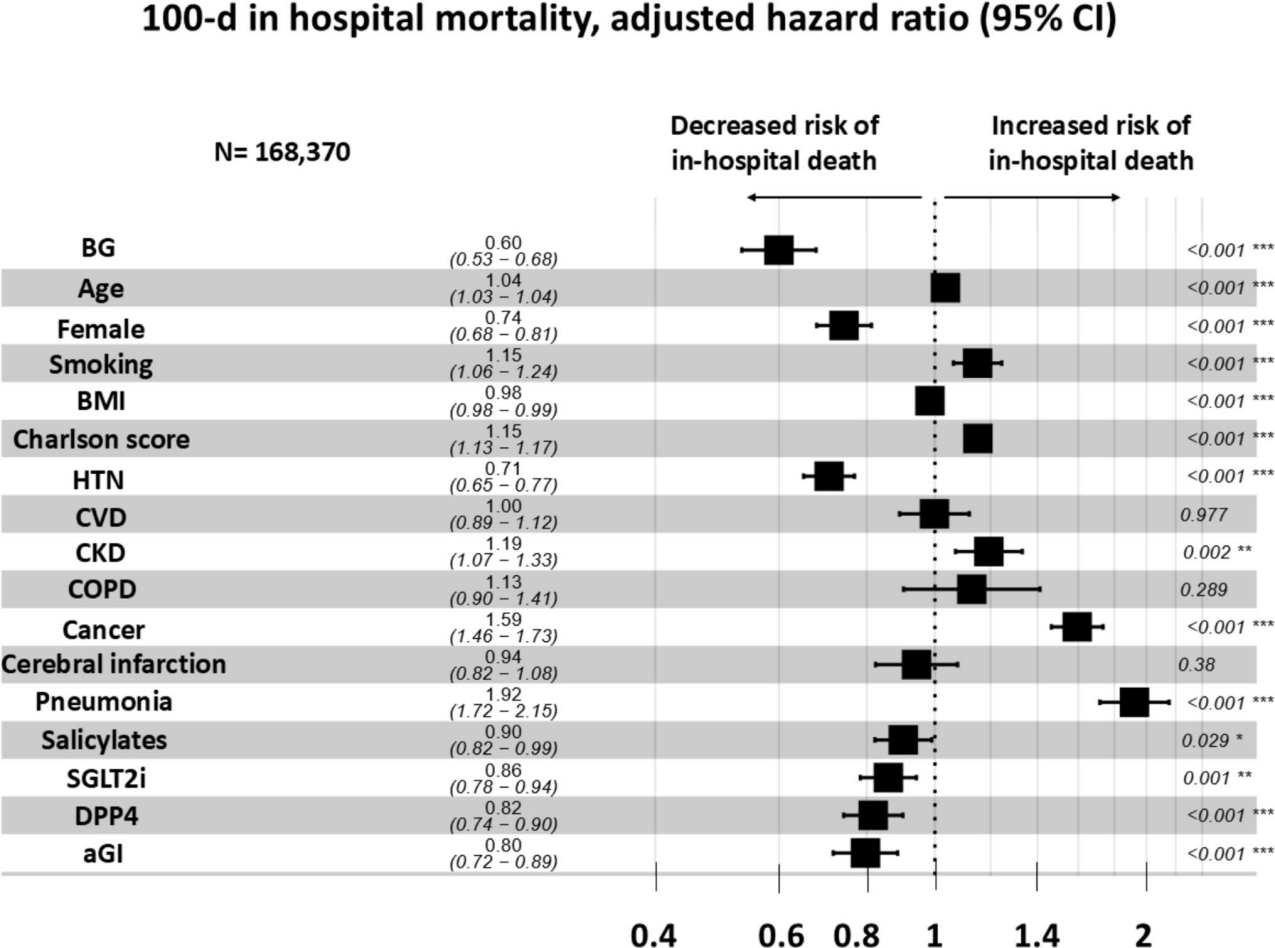
A forest plot showing the hazard ratio and 95% confidence intervals associated with variables with time to the primary endpoint (100-day in-hospital death). Black squares represent the hazard ratios, and the horizontal bars extend from the lower limit to the upper limit of the 95% confidence interval of the hazard ratio estimate. *BG* biguanide, *BMI* body mass index; *HTN* hypertension, *CVD* cardiovascular disease, *CKD* chronic kidney disease, *COPD* chronic obstructive pulmonary disease, *SGLT2i* sodium–glucose cotransporter type 2 inhibitors (SGLT2s), *DPP4* dipeptidyl peptidase-4 inhibitors, *aGI* alpha-glucosidase inhibitors

In the Kaplan–Meier analysis, patients with COVID-19 with BG treatment had a significantly higher survival rate compared with those without BG treatment (*P* < 0.001, Log-rank chi-square = 155) (Fig. 2). Cox proportional hazards model analysis also showed that BG therapy was associated with a reduction in mortality (HR, 0.599; 95% CI, 0.531–0.677; *P* < 0.001) (Fig. 3).

### In-hospital BG use is associated with lower incidence rate of AKI in admitted patients with COVID-19 under unmatched cohort

AKI developed in 1746 of 168,370 patients (1.04%). In the univariate, unadjusted analysis, patients receiving BG were less likely to experience the secondary endpoint event compared with those who did not (odds ratio, 0.59; 95% CI, 0.504–0.676) (Table 3). In multivariate logistic regression analyses, BG use was significantly associated with a lower AKI onset in all models (Table 3). The severity of chronic kidney disease (CKD) is a key confounding factor in the development of acute kidney injury (AKI), but it is difficult to accurately assess CKD severity using the DPC database due to limited detailed information. Therefore, to confirm the potential protective effect of BG on AKI outcomes, we conducted a multivariate logistic regression analysis restricted to patients without a CKD diagnosis (n = 153,574) as a sensitivity analysis. Consistent with the primary results, BG use was associated with a reduced odds of AKI in this non-CKD population (adjusted odds ratio: 0.552; 95% CI: 0.463–0.654; *P* < 0.001). (Supplementary Table 3).

**Table 3:** Association of BG use and AKI onset in the univariate and multivariable analyses in the overall study cohort

	Unadjusted OR	*P*	Model 1^a^		Model 2^b^	
			Adjusted OR	*P*	Adjusted OR	*P*
	(95% CI)		(95% CI)		(95% CI)	
**Biguanide treatment,**	**0.59**	** < 0.001**	**0.60**	** < 0.001**	**0.55**	** < 0.001**
**yes (ref = no)**	**(0.504, 0.676)**		**(0.513, 0.689)**		**(0.505, 0.681)**	
Age (per 1-year increase)			1.00	0.858	1.00	0.923
			(0.995, 1.004)		(0.995, 1.006)	
Female sex			0.82	< 0.001	0.80	0.001
(ref = male)			(0.728, 0.914)		(0.702, 0.913)	
Smoking			1.01	0.877	1.04	0.508
(ref = no)			(0.911, 1.117)		(0.925, 1.172)	
BMI			0.99	0.329	1.00	0.384
(per increase of 1)			(0.985, 1.005)		(0.994, 1.011)	
Charlson score			1.05	< 0.001	1.07	0.002
(per 1-year increase)			(1.012, 1.077)		(1.024, 1.109)	
Pneumonia,					1.24	0.067
yes (ref = no)					(0.978, 1.553)	
Salicylates treatment					1.05	0.489
yes (ref = no)					(0.906, 1.223)	
**SGLT2 treatment,**					0.73	< 0.001
**yes (ref = no)**					(0.632, 0.832)	
**DPP4 treatment,**					1.06	0.402
**yes (ref = no)**					(0.922, 1.229)	
**αGI treatment,**					1.02	0.821
**yes (ref = no)**					(0.871, 1.184)	

^a^Model 1 adjusted for age, sex, history of smoking, BMI and Charlson score

^b^Model 2 adjusted for all variables in model 1 plus comorbidities of hypertension, cardiovascular disease, chronic kidney disease (CKD), chronic obstructive pulmonary disease, malignancy, cerebral infarction (CeI), pneumonia and use of salicylates, vasopressors, SGLT2 inhibitors, DPP4 inhibitors, aGI treatment and insulin use

### BG usage is associated with lower in-hospital mortality rates and lower incidence rate of AKI in admitted patients with COVID-19 under propensity score-matched cohort

A major limitation in retrospective studies is the bias in the likelihood of patients receiving the treatments studied. In unadjusted observational studies, disease severity is a confounding factor that affects treatment decisions and outcomes, often precluding accurate analysis of potential treatment effects. Thus, propensity score matching for disease severity and other variables has been used in some observational studies, leading to findings compatible with those obtained from randomised controlled trials [19, 20]. In the clinical setting, some patients may not receive BG due to lactic acidosis risk, particularly with metformin, known as metformin-associated lactic acidosis (MALA) [21]. Although MALA is an extremely rare condition, MALA can be induced by several conditions that either increase lactate production or decrease its clearance, including renal impairment, liver disease, heart failure and malignancy. Tumours can produce large amounts of lactate through anaerobic glycolysis, known as the Warburg effect [22]. Thus, we used propensity score matching and multivariable regression analysis incorporating markers of disease severity and other clinical covariates, including age, sex, BMI, smoking, Charlson score, comorbidities of malignancy, CKD, cardiovascular disease, pneumonia, COPD, use of anti-diabetes medications (i.e. DPP4 inhibitors, SGLT2 inhibitors and aGI), and insulin use (see also Supplementary Method). Accordingly, the groups exposed and not exposed to BG had 30,908 patients in each group. The differences between BG and clinical co-variables were attenuated in the propensity score-matched samples compared with the unmatched samples (Table 4**, **Supplementary Fig. 2).

**Table 4:** Characteristics of patients receiving or not receiving BG after propensity score matching

Propensity matched data	All (*n* = 61,816)	No biguanide (*n* = 30,908)	Biguanide (*n* = 30,908)	
Variables				Absolute standardised difference
Patient demographics				
Age (SD)	65.5 (11.9)	65.2 (12.5)	65.9 (11.3)	0.06
Male sex, n (%)	40,927 (66.2)	20,480 (66.3)	20,447 (66.2)	0.001
BMI (kg/m^2^)	25.5 (5.67)	25.6 (6.16)	25.5 (5.13)	0.03
Smoking, n (%)	16,164 (52.3)	16,131 (52.2)	16,164 (52.3)	0.001
Charlson score (SD)	0.78 (1.32)	0.81 (1.33)	0.75 (1.31)	0.04
Comorbidities				
Hypertension (%)	23,574 (38.1)	11,814 (38.2)	11,760 (38.0)	–
Malignancy, *n* (%)	14,294 (23.1)	7130 (23.1)	7164 (23.2)	0.001
CKD, *n* (%)	777 (1.26)	413 (1.34)	364 (1.18)	0.002
Cardiovascular disease, *n* (%)	8276 (13.4)	4228 (14.0)	3938 (12.7)	0.01
Cerebral infarction, *n* (%)	4932 (7.98)	2216 (7.17)	2716 (8.79)	–
Pneumonia, *n* (%)	2469 (3.99)	1274 (4.12)	1195 (3.87)	0.002
COPD, *n* (%)	867 (1.40)	430 (1.39)	437 (1.41)	0.0002
Other medications				
DM meds				
DPP4, *n* (%)	37,694 (60.9)	18,575 (60.1)	19,099 (61.8)	0.017
SGLT2, *n* (%)	20,817 (33.7)	11,188 (36.2)	9629 (31.2)	0.05
SU, *n* (%)	7496 (12.1)	3988 (12.9)	3508 (11.3)	–
aGI, *n* (%)	7741 (12.5)	4172 (13.5)	3569 (11.5)	0.02
GLP1, *n* (%)	2436 (3.94)	1692 (5.47)	744 (2.41)	–
TZD, *n* (%)	2120 (3.43)	516 (1.67)	1604 (5.19)	–
Salicylate, *n* (%)	14,060 (22.7)	7083 (22.9)	6977 (22.6)	–
Insulin use, *n* (%)	35,041 (56.7)	17,642 (57.1)	17,399 (56.3)	0.007

Normally distributed variables are presented as mean ± standard deviation (SD). Categorical data are presented as counts and percentages. *BMI* body mass index, *CKD* chronic kidney disease, *COPD* chronic obstructive pulmonary disease, *DM* diabetes mellitus, *DPP4* dipeptidyl peptidase-4 inhibitors, SGLT2i sodium–glucose cotransporter type 2 inhibitors, *SU* sulfonylureas, *aGI* alpha-glucosidase inhibitors, *GLP-1* glucagon-like peptide-1 analogues, *TZD* thiazolidinediones

**Fig. 4:**
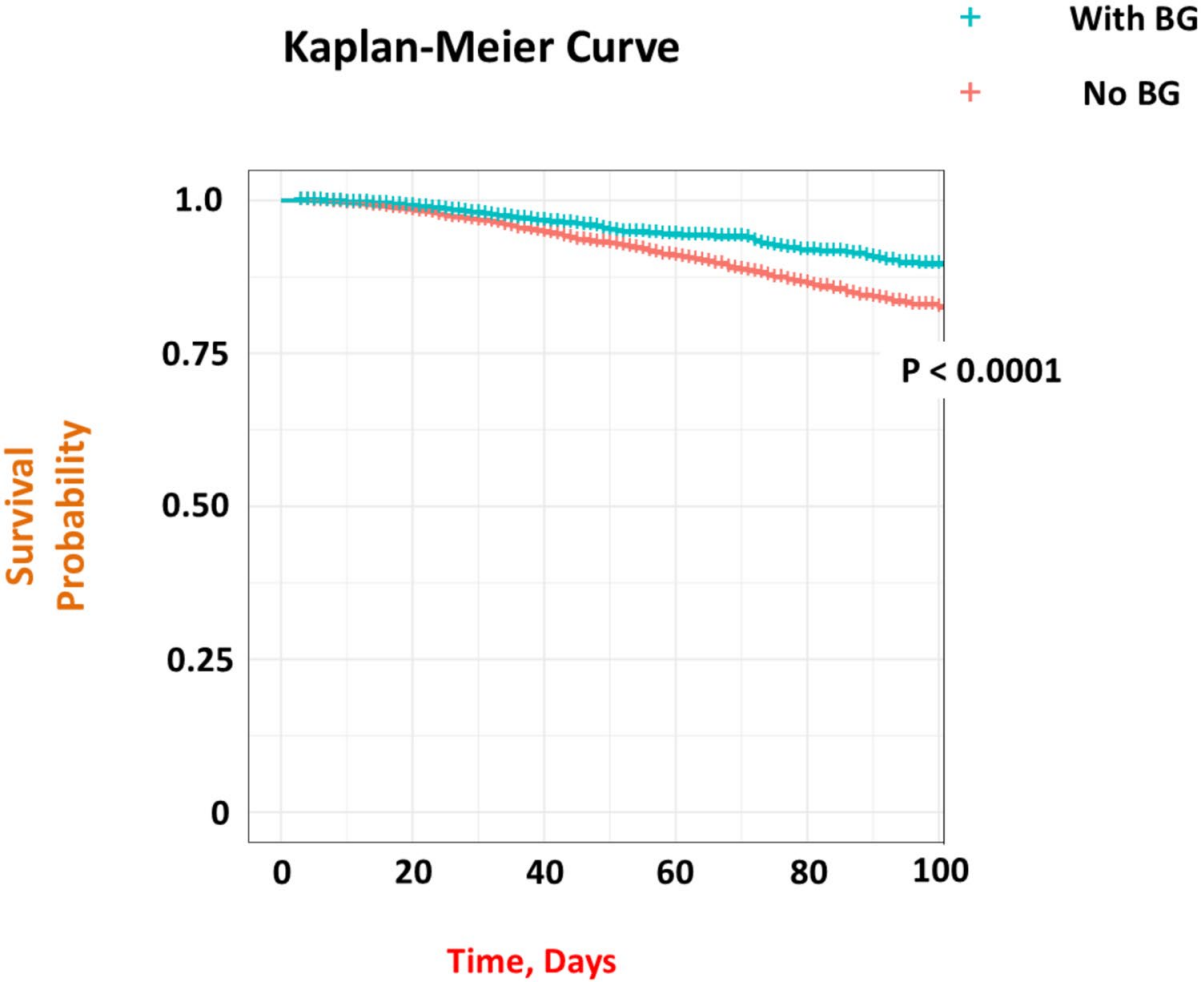
Kaplan–Meier estimates of all-cause mortality for admitted patients with COVID-19 with and without biguanide use under propensity score-matched cohort. Patients with COVID-19 treated with BG had a statistically significant higher survival rate than those not treated with BG (*P* < 0.001, Log-rank Chi-square = 64.4) even in the propensity score-matched cohort. *PS* propensity score, *BG* biguanide

Subsequently, using this propensity score-matched group of 61,816 patients, we explored the effects of in-hospital BG use. From logistic regression analysis, patients receiving BG were less likely to have had a primary endpoint event than those who did not (odds ratio, 0.611; 95% CI, 0.537–0.693; *P* < 0.001). Cumulative incidence curves also showed significantly reduced in-hospital death amongst propensity score-matched patients treated with BG (Fig. 4). Cluster-paired Cox proportional hazards regression model analysis also showed that BG therapy was associated with a reduction in mortality (hazard ratio, 0.619; 95% CI, 0.545–0.702; *P* < 0.001). Regarding the secondary endpoint of AKI onset, logistic regression analysis showed that patients receiving BG were less likely to have had a secondary endpoint event than those who did not (odds ratio, 0.554; 95% CI, 0.465–0.655). As a sensitivity analysis, based on the rationale described above, a propensity score-matched analysis was conducted in the population limited to non-CKD patients. Consistent with the previous results, BG use was significantly associated with a lower incidence of AKI in the non-CKD population (odds ratio: 0.578; 95% CI: 0.486–0.687), which aligns with the findings from the multivariable logistic regression analysis.

## Discussion

In our nationwide study of hospitalised patients with DM-COVID-19 in Japan, we showed that in-hospital biguanide use was significantly associated with decreased mortality and a lower incidence rate of AKI. In addition to the multivariable regression analysis for unmatched cohort, we used propensity score matching and multivariable regression analysis to diminish treatment selection bias by generating treatment and control groups with well-balanced covariates, allowing a more reliable comparison of potential biguanide treatment effects. Our findings indicated a beneficial role for biguanide in treating hospitalised patients with COVID-19. To the best of our knowledge, this is the first observational study indicating an association between biguanide use and decreased rate of AKI development in hospitalised patients with COVID-19.

The emergence of SARS-CoV-2 created a worldwide public health emergency. In December 2019, SARS-CoV-2, the causative agent of COVID-19, was first isolated during an outbreak in Wuhan, China [1]. In March 2020, the World Health Organisation (WHO) declared COVID-19 a pandemic due to its rapid dissemination to several countries and continents. Since January 2020, COVID-19 has caused almost 800 million cases of the disease worldwide and over 7 million deaths (https://covid19.who.int). This is 100,000 times as many cases as SARS in 2003 and 10,000 times as many deaths. Currently, approximately 30% of the world population, including 70% of people in low-income countries, remain unvaccinated against COVID-19 [23]. Although the WHO ended its public health emergency for COVID-19 on 11th May, 2023, COVID-19 remains a pandemic as of July 2024 [24].

Although several anti-viral agents, including nirmatrelvir/ritonavir, molnupiravir, azvudine and remdesivir, are approved as COVID-19 therapeutics (https://www.fda.gov/drugs/emergency-preparedness-drugs/coronavirus-covid-19-drugs), there are challenges in ensuring global access to these agents, particularly in developing countries. This is due to regulatory barriers, economic constraints and other obstacles. Therefore, new, safer and more efficient COVID-19 treatments should be discovered that are accessible and affordable to help combat and ultimately end the pandemic. Drug repurposing is an essential strategy because it can save a considerable amount of time and money because the pharmacokinetics, pharmacodynamics and safety profiles of these drugs are already established [25, 26].

The biguanide metformin is the most prescribed drug for approximately 150 million individuals with type 2 diabetes worldwide. Several studies identified metformin as a potential therapeutic agent because of its possible action against proteins involved in the mRNA translation process, anti-viral activity in vitro and anti-inflammatory and antithrombotic activities [27, 28]. However, the therapeutic effect of metformin for COVID-19 remains controversial. Although several observational studies have suggested that patients with COVID-19 receiving metformin for diabetes had a lower risk of progressing to severe COVID-19 [14, 29], some clinical trials (TOGETHER trial, COVID-OUT trial) implied that metformin use did not reduce the risk of hospitalisation or death [30, 31]. This discrepancy may be attributed to the complex interplay of factors influencing the prognosis of COVID-19, including the adequacy of medical infrastructure, the availability of healthcare personnel, and variations in patient demographics [15, 16]. Our result is consistent with a recent meta-analysis examining the relationship between metformin use and in-hospital mortality, which included 22 studies—all conducted outside of Japan [32] (Supplementary Table 4). Therefore, our study provides a valuable contribution to the existing body of evidence by evaluating the potential effects of biguanides on mortality in COVID-19 patients within the Japanese context. Furthermore, as shown in Supplementary Table 4**,** our study includes the largest patient population amongst the 22 studies in the meta-analysis, which represents a key strength of this research. Our results also suggest lower mortality amongst females compared to males in the context of COVID-19, which is consistent with previous findings [33]. Differences in sex hormone levels may modulate the immune response and reduce COVID-19 severity in females relative to males, warranting further investigation.

The hypoglycemic effects of metformin are well-known, but recent attention has been given to its additional effects, such as its potential to protect the kidneys through the activation of AMP-activated protein kinase (AMPK) in the renal tubular epithelium [34, 35]. AMPK activity is exquisitely sensitive to cellular energy stress, reflected in the increasing AMP level and increased AMP–adenosine triphosphate (ATP) ratio, indicating that AMPK is a critical cellular energy sensor [36]. Because the kidneys consume a lot of energy to regulate body fluids and blood pressure and to excrete waste products and toxins, AMPK dysregulation leads to impaired renal tubular homeostasis [37]. We recently reported that dysregulation of the AMPK signal in the proximal tubule induced tubular atrophy and fibrosis in the subtotal nephrectomy mouse model [38][38]. In addition, AMPK has been found to directly affect virus entry through the modification of the viral receptor angiotensin-converting enzyme 2 (ACE2) [40]. AMPK modifies ACE2 by phosphorylating ACE2 at Ser680 in human endothelial cells from umbilical vein and human embryonic kidney 293 (HEK293T) cells [41]. Several kidney-specific RNA-Seq datasets provide detailed information about ACE2 gene expression in kidney cell types, indicating that ACE2 mRNA is strongly expressed in proximal tubule epithelial cells [42, 43] but not in more distal renal tubule cells, podocytes, mesangial cells or glomerular endothelial cells [44]. Thus, AMPK induces a conformational change in the ACE2 receptor in the proximal tubular cells, resulting in decreased binding affinity with the SARS-CoV-2, potentially reducing infectivity and the risk of severe disease [45]. The mechanisms underlying metformin’s reno-protective effects for COVID-19-induced renal damage should be clarified in future studies.

In our study, the average daily dose of metformin was approximately 785 mg, with a median dose of around 750 mg (Supplementary Fig. 1). Although stronger evidence for mortality reduction with biguanides has been observed at higher doses [46] in the European outpatient population, we believe that a 750 mg dose may still provide a mortality benefit for hospitalised patients in Asian population. This is particularly relevant for patients who often receive reduced or temporarily discontinued metformin due to acute illnesses, such as COVID-19, because of safety concerns and altered pharmacokinetics [46, 47].

This study has several limitations. First, laboratory measures of diabetes mellitus severity, such as HbA1c and in-hospital blood-glucose levels, were not available in the DPC system. Consequently, we could not accurately assess the severity of diabetes, although we extracted information on insulin use to approximate it as best as possible. Second, the study population was limited to Japanese patients, which may restrict the generalizability of the findings to other populations or ethnic groups. Third, the proportion of patients prescribed biguanides was relatively low (18.4%), which is lower than the reported rate of metformin prescriptions in the general population [48]. This discrepancy, possibly due to the inclusion of only hospitalised patients [49], may introduce selection bias. Fourth, the DPC system lacks comprehensive and accurate documentation of medical history, which may have contributed to unexpected associations—such as the observed decreased odds of in-hospital mortality in patients with complications like hypertension or cerebral infarction, as shown in Fig. 3. Fifth, nearly all biguanide prescriptions in this study were for metformin (99.7%), whilst buformin accounted for only 0.3%, indicating that the findings primarily reflect the effects of metformin. Finally, as this was an observational study, causality cannot be established.

In summary, this is the first study showing that in-hospital BG use affects in-hospital mortality and incidence rate of AKI in patients with COVID-19 in Japan. These findings provide a foundation for future studies to clarify the mechanisms of metformin’s reno-protective role in patients with COVID-19 patients.

## Supplementary Information

Below is the link to the electronic supplementary material.

Supplementary file1 (DOCX 36924 KB)

## Data Availability

The data are not publicly available since the information present in this study could compromise the privacy of research participants. The data that support the findings of this study are available from the corresponding author, Hiroaki Kikuchi, upon reasonable request. All other relevant data are within the manuscript and its Supplemental Information files.
