# Supplementary material for: Biguanides are associated with decreased early mortality and risk of acute kidney injury in hospitalised patients with COVID-19: a nationwide retrospective cohort study in Japan
Source: Clin Exp Nephrol. 2025 Aug 27;30(1):33–44. doi: 10.1007/s10157-025-02755-z (PMC12811280; doi:10.1007/s10157-025-02755-z)
Supplement: Supplementary file 1 — Supplementary file1 (DOCX 36924 KB) [file 10157_2025_2755_MOESM1_ESM.docx]

**Biguanides are associated with decreased early mortality and risk of**

**acute kidney injury in hospitalised patients with COVID-19**

**: A nationwide retrospective cohort study in Japan**

**Supplementary method:**

**Statistical analysis**

Data acquisition and analysis were performed using R, version 4.0.2 (R Foundation for Statistical Computing, Vienna, Austria). Normally distributed variables were presented as the mean ± standard deviation (SD). Categorical data were presented as counts and percentages. Intergroup comparisons were performed using the t-test or χ^2^ test, as appropriate. The primary analysis of interest was the relationship between BG use and all-cause mortality. Multivariate logistic regression analysis was performed to identify variables independently associated with the primary outcome. The collinearities of candidate variables in the multivariate logistic regression analysis were evaluated using the Pearson product-moment correlation coefficients (Pearson score). Kaplan–Meier survival curves were constructed, and log-rank testing was performed to assess the time to event of the primary outcome. A Cox proportional hazards model was used to evaluate the association between the BG groups and primary outcome. The multivariate Cox proportional hazards models were adjusted for the demographics, comorbidities and medications for DM other than BG. The results were shown as hazard ratios (HRs) and 95% confidence intervals (CIs).

Propensity score matching was performed to balance covariates between patients treated with BG and those without. Multivariable logistic regression models were used to calculate propensity scores. Propensity scores included covariates that may affect the likelihood of patients receiving the treatment of interest and the outcome of interest, as well as unbalanced covariates between treatment groups before matching. Matching based on propensity scores was performed using a 1:1 nearest-neighbour algorithm, with a calliper width of 0.20.

**Data extraction from DPC system**

For the present study we used the Japanese DPC Database(1), which is a patient discharge and administrative claims data base to which the acute care hospitals under the DPC reimbursement policy voluntarily submit. The DPC database is similar to the US Medicare claims database (2) or the US Nationwide Impatient Samples (3). The DPC has been conducted by the DPC Research Group funded by the Ministry of Health, Labour and Welfare, Japan. As of April 2020, the payment system initially introduced to 82 hospitals in 2003 has been applied to 1,757 hospitals with a total of 483,180 beds. This number is thought to be enough to cover almost all acute inpatients and is about 30% of all hospitals with beds for general patients (including those in subacute care and rehabilitation, but excluding those with mental illness, infectious disease, tuberculosis, and long-term care) and about 54% of all beds of hospitals with beds for general patients across the country (1). The DPC database can be used to identify, track, and analyze national trends in health-care utilization, access, quality, outcomes, and costs. The database includes the following information: location of hospital; patient age and sex; diagnoses, comorbidities at admission and complications after admission coded with International Classification of Diseases, 10th Revision codes; procedures coded according to the Japanese claim classification (K-codes); drugs and devices used; length of stay (LOS); in-hospital mortality; and costs. The diagnoses are recorded by the physicians in charge with reference to the medical charts.

We selected all the DPC hospitals that participated in the DPC survey every year between 2021 and 2023 which cover the era following the development of COVID-19 vaccines. In Japan, vaccination for COVID-19 was introduced in February 2021. All patients who were diagnosed with COVID-19 infection (DPC 2013 code B-342) were enrolled in the present study.

**Statistical methods used**

Comparison of baseline population characteristics with continuous variables (**Table 1**) including age, BMI, Charlson score are analyze by unpaired- T test using t.test () function in R. Comparison of baseline population characteristics with categorical variables (**Table 1**) including Sex, Smoking, Hypertension, Malignancy, Chronic kidney disease, Cardiovascular disease, Cerebral infarction, Pneumonia, COPD, DM-meds (DPP4, SGLT2, SU, aGI, GLP1, TZD) , Salicylate were analyzed by chi-squared test using chisq.test () function in R. Association of BG usage and the primary outcome and secondary outcome (**Table 2,3, Supplementary Table 3**) were analyzed by logistic regression analysis using glm () function in R. Kaplan–Meier analysis was performed using survfit () function. Logrank test was performed using Surv () function in R. Cox proportional hazard model analysis was performed using coxph () function in R. Propensity Score Matching (**Table 4**) was performed using a 1:1 nearest-neighbor algorithm, with a caliper width of 0.20 using matchit () function in R.


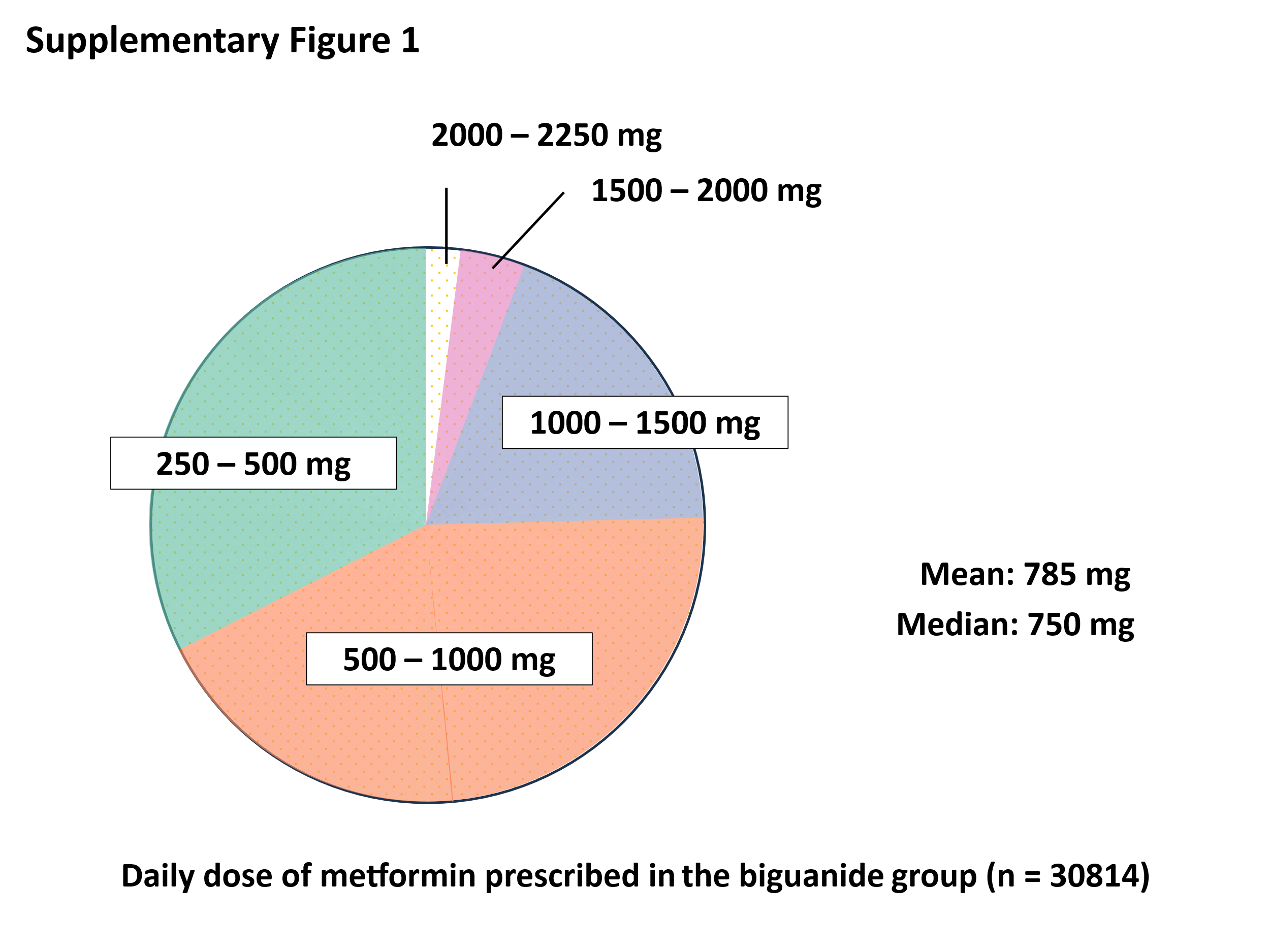
**Supplementary Figure**

**Supplementary Figure 1.** Distribution of daily metformin doses in the biguanide group


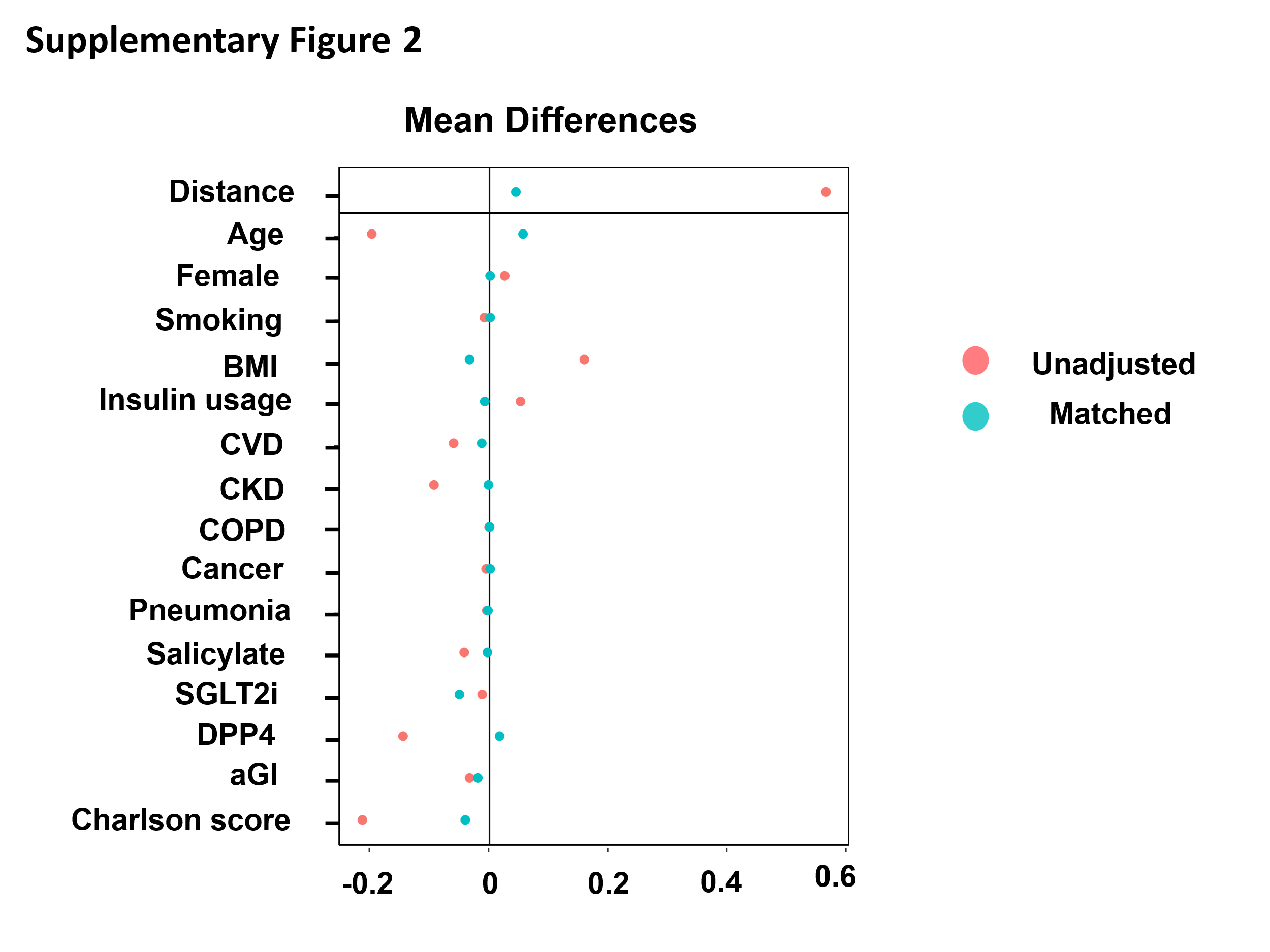


**Supplementary Figure 2. Standardized mean differences in the unmatched and matched sample**

**Supplementary Table**

**Supplementary Table 1. ICD-10 codes used for this study**

| ***Diseases*** | **ICD-10 codes (2013 version)** | | | | | | | | | |
| --- | --- | --- | --- | --- | --- | --- | --- | --- | --- | --- |
| **Chronic kidney disease** | N18.0 | N18.8 | N18.9 | N19 |  |  |  |  |  |  |
| **Acute kidney injury** | N17.0 | N17.1 | N17.2 | N17.8 | N17.9 |  |  |  |  |  |
| **Diabetes Mellitus** | E10.X | E11.X | E12.X | E13.X | E14.X |  |  |  |  |  |
| **Hypertension** | I10 | I11.X | I12.X | I13.X | I15.X |  |  |  |  |  |
| **Cardiovascular Disease** | I20.X | I21.X | I22.X | I23.X | I24.X | I25.X |  |  |  |  |
| **Solid Malignancy** | C00.X | C01 | C02.X | C03.X | C04.X | C05.X | C06.X | C07.X | C08.X | C09.X |
|  | C10.X | C11.X | C12 | C13.X | C14.X | C15.X | C16.X | C17.X | C18.X | C19 |
|  | C20 | C21.X | C22.X | C23 | C24.X | C25.X | C26.X |  |  |  |
|  | C30.X | C31.X | C32.X | C33 | C34.X | C37 | C38.X | C39.X |  |  |
|  | C40.X | C41.X | C43.X | C44.X | C45.X | C46.X | C47.X | C48.X | C49.X |  |
|  | C50.X | C51.X | C52 | C53.X | C54.X | C55 | C56 | C57.X | C58 |  |
|  | C60.X | C61 | C62.X | C63.X | C64.X | C65 | C66 | C67.X | C68.X | C69.X |
|  | C70.X | C71.X | C72.X | C73.X | C74.X | C75.X |  |  |  |  |
|  | D00.X | D01.X | D02.X | D03.X | D04.X | D05.X | D06.X | D07.X | D09.X |  |
| **Non-solid malignancy** | C81.X | C82.X | C83.X | C84.X | C85.X | C88.X | C89 |  |  |  |
|  | C90.X | C91.X | C92.X | C93.X | C94.X | C95.X | C96.X |  |  |  |
| **Cerebral Infarction** | I63.X | I66.X |  |  |  |  |  |  |  |  |
| **Pneumonia** | J15.X | J18.X | J84.X |  |  |  |  |  |  |  |
| **Chronic obstructive pulmonary disease** | J44.X |  |  |  |  |  |  |  |  |  |
| **COVID-19 infection** | **B34.2** |  |  |  |  |  |  |  |  |  |

**Supplementary Table 2. Receipt numbers used for this study**

| ***Treatment*** | **Receipt numbers for treatment** | | | | | | | | | |
| --- | --- | --- | --- | --- | --- | --- | --- | --- | --- | --- |
|  |  |  |  |  |  |  |  |  |  |  |
| **Insulin** | 620000265 | 620000266 | 620000269 | 620000448 | 620002439 | 620002440 | 620008894 |  |  |  |
|  | 620008895 | 620008896 | 620008897 | 620008907 | 620008909 | 620008910 | 620008912 |  |  |  |
|  | 620008913 | 620008915 | 620008916 | 620008943 | 620008945 | 620008952 | 620008953 |  |  |  |
|  | 621911101 | 621911201 | 621911301 | 621973201 | 622198901 | 622199001 | 622451001 |  |  |  |
|  | 629905601 | 629905701 | 629905801 | 629906701 | 629906801 | 629906901 | 629907001 |  |  |  |
|  | 629907301 | 629907401 | 629907501 | 640451027 | 640453023 |  |  |  |  |  |
| **Biguanide (BG)** |  |  |  |  |  |  |  |  |  |  |
| **metformin-250** | 621974701 | 620004480 | 610444147 | 622450301 | 621986301 |  |  |  |  |  |
| **metformin-500** | 622242501 | 622450401 | 622517101 |  |  |  |  |  |  |  |
| **Buformin** | 620004502 | 613960014 | 613960015 |  |  |  |  |  |  |  |
| **Salicylate** | 610407146 | 610408009 | 610409338 | 610421011 | 610431009 | 610441006 | 610443053 | 610443056 | 610443057 | 610463004 |
|  | 611140017 | 611140019 | 611140181 | 611140793 | 611140794 | 611140795 | 611140798 | 611140849 | 611140850 | 616210018 |
|  | 616220021 | 620000065 | 620000483 | 620000484 | 620000485 | 620000486 | 620000487 | 620000488 | 620001562 | 620001612 |
|  | 620001952 | 620002629 | 620004280 | 620004727 | 620004897 | 620008070 | 620008510 | 620008624 | 620008997 | 620506801 |
|  | 621362001 | 621375001 | 621867201 | 621969902 | 622023001 | 622173901 | 622234801 | 622341601 | 622442701 | 622517001 |
|  | 641140027 | 641140028 | 641140079 | 641140113 | 660408074 | 660408076 | 660421082 | 660441023 | 660470002 | 662640125 |
|  | 662650001 | 662650002 | 662650007 | 662650015 | 662650092 | 662650093 | 662650108 | 662660006 | 666210001 | 810000000 |
| **Thiazolidinediones (TZD)** | 610432040 | 610432041 | 621990901 | 621991001 | 621986301 | 621986301 | 622048401 | 622048501 | 622086101 | 622086001 |
| **Sulfonylureas (SU)** | 620006030 | 613960017 | 620009209 | 620002032 | 620002031 | 620003947 | 620003948 | 613960002 | 613960003 | 620003159 |
|  | 620003160 | 610443002 | 610443003 | 621982701 | 622122201 | 622122301 | 622217701 | 610432032 | 610432033 | 610432026 |
|  | 610432027 | 620001907 | 620001908 | 622462501 | 622040901 | 622041001 |  |  |  |  |
| **Sodium-glucose cotransporter type 2 inhibitors (SGLT2)** | 622306601 | 622306701 | 622341901 | 622342001 | 622335701 | 622335801 | 622336801 | 622340101 | 622360601 | 622401201 |
|  | 622401301 |  |  |  |  |  |  |  |  |  |
| **Dipeptidyl peptidase-4 inhibitors (DPP-4)** | 621950901 | 621951001 | 621951101 | 622277501 | 621970601 | 621970701 | 621970801 | 622288401 | 621980701 | 621986101 |
|  | 621986201 | 621986001 | 622093501 | 622660601 | 622182601 | 622201701 | 622245601 | 622245701 | 622415401 |  |
| **alpha-glucosidase inhibitors (aGI)** | 610406390 | 610406391 | 620002120 | 620002121 | 620005563 | 620002841 | 620002843 | 620002845 | 620002847 | 620004069 |
|  | 620004070 | 620004071 | 620004072 | 620004073 | 620004074 | 620005557 | 620005558 | 620005559 | 620005560 | 620005561 |
|  | 620005562 | 620005564 | 620005565 | 620005566 | 620008071 | 620008072 | 620008073 | 620008074 | 620008075 | 620008076 |
|  | 620008726 | 620008728 | 621665301 | 621665401 | 621673501 | 621673601 | 621683401 | 621683501 | 621689001 | 621689101 |
|  | 621689303 | 621689403 | 621690203 | 621690303 | 621690402 | 621690502 | 621690901 | 621691001 | 621691201 | 621691601 |
|  | 621943301 | 621943401 | 622090001 | 622090101 | 622662501 | 622662601 | 622662701 | 622662801 | 613960081 | 613960082 |
|  | 621937201 | 621937101 | 621942102 | 621942202 | 620009288 | 620009289 | 620009290 | 620009291 | 620009292 | 620009293 |
|  | 620009294 | 620009295 | 620009296 | 620009297 | 621784902 | 621785002 | 621896402 | 621896502 | 620003127 | 620003128 |
|  | 620003129 | 622432501 | 622426601 | 622426701 | 622560201 | 622628601 | 622560301 | 622628701 | 622560401 | 622628801 |
|  | 622664101 | 622544301 | 622664201 | 622544401 | 622664301 | 622544501 |  |  |  |  |
| **Glucagon-like peptide-1 (GLP-1) analogs** | 621974801 | 622038301 | 622038401 | 622406001 |  |  |  |  |  |  |

**Supplementary Table 3. Association of BG use and AKI onset in the univariate and multivariable analyses in the non-CKD patients (n= 153,574).**

|  |  | |  | Model 1^a^ | | Model 2^b^ | |
| --- | --- | --- | --- | --- | --- | --- | --- |
|  | | Unadjusted OR | *P* | Adjusted OR | *P* | Adjusted OR | *P* |
|  |  | (95% CI) |  | (95% CI) |  | (95% CI) |  |
|  | |  |  |  |  |  |  |
| **Biguanide treatment,** | | **0.58** | **<0.001** | **0.59** | **<0.001** | **0.55** | **<0.001** |
| **yes (ref = no)** | | **(0.499, 0.672)** |  | **(0.507, 0.683)** |  | **(0.463, 0.654)** |  |
|  | |  |  |  |  |  |  |
| Age (per 1-year increase) | |  |  | 1.00 | 0.858 | 1.00 | 0.872 |
|  |  |  |  | (0.995, 1.004) |  | (0.995, 1.006) |  |
| Female sex | |  |  | 0.80 | <0.001 | 0.79 | <0.001 |
| (ref = male) | |  |  | (0.713, 0.906) |  | (0.691, 0.910) |  |
| Smoking | |  |  | 1.02 | 0.877 | 1.06 | 0.367 |
| (ref = no) | |  |  | (0.915, 1.133) |  | (0.935, 1.199) |  |
| BMI | |  |  | 1.00 | 0.329 | 1.00 | 0.185 |
| (per increase of 1) | |  |  | (0.988, 1.007) |  | (0.996, 1.012) |  |
| Charlson Score | |  |  | 1.05 | <0.001 | 1.08 | <0.001 |
| (per 1-year increase) | |  |  | (1.018, 1.085) |  | (1.038, 1.127) |  |
| Pneumonia, | |  |  |  |  | 1.19 | 0.159 |
| yes (ref = no) | |  |  |  |  | (0.925, 1.516) |  |
| Salicylates treatment | |  |  |  |  | 1.12 | 0.161 |
| yes (ref = no) | |  |  |  |  | (0.954, 1.309) |  |
| SGLT2 treatment, | |  |  |  |  | 0.68 | <0.001 |
| yes (ref = no) | |  |  |  |  | (0.590, 0.784) |  |
| DPP4 treatment, | |  |  |  |  | 1.02 | 0.819 |
| yes (ref = no) | |  |  |  |  | (0.879, 1.181) |  |
| αGI treatment, | |  |  |  |  | 1.09 | 0.310 |
| yes (ref = no) | |  |  |  |  | (0.924, 1.270) |  |

^a^ Model 1 adjusted for age, sex, history of smoking, BMI and Charlson Score.

^b^ Model 2 adjusted for all variables in model 1 plus comorbidities of hypertension, cardiovascular disease, chronic kidney disease (CKD), chronic obstructive pulmonary disease, malignancy, cerebral infarction (CeI), pneumonia and use of salicylates, vasopressors, SGLT2 inhibitors, DPP4 inhibitors, aGI treatment and Insulin use.

**Supplementary Table 4. Summary of previous studies** (4) **reporting associations between BG and in-hospital mortality along with findings from our study**

|  | Study | year | country | # of participants | mean follow up period, max follow up (days) | study period | OR | HR |
| --- | --- | --- | --- | --- | --- | --- | --- | --- |
| 1 | PMID: 32409498 | 2020 | China | 120 | 23-24, N/A | 2020/01/01-2020/03/17 | N/A | (MET: 9.3%, w\o MET): 19.5% |
| 2 | PMID: 33471718 | 2020 | China | 131 | 17.3, 60 | 2020/01/23-2020/03/19 | 0.2 | 0.22 |
| 3 | PMID: 34670765 | 2021 | China | 991 | N/A ,N/A | 2020/01/23-2021/02/28 | 0.33 | 0.51 |
| 4 | PMID: 33310173 | 2021 | China | 328 | N/A, 30 | 2019/12/31-2020/03/31 | N/A | 0.54 |
| 5 | PMID: 34585841 | 2022 | China | 131 | N/A, 50 | 2020/01/23-2020/03/19 | 0.19 | N/A |
| 6 | PMID: 32446312 | 2020 | China | 283 | 19.5-21.0, N/A | 2020/01/27-2020/03/24 | 0.21 | N/A |
| 7 | PMID: 32861268 | 2020 | China | 1213 | 21, 28 | NA | N/A | 1.65 |
| 8 | PMID: 33580540 | 2021 | USA | 9555 | N/A, 45 | 2020/03/04-2020/12/04 | 0.32 | N/A |
| 9 | PMID: 33521772 | 2021 | USA | 6256 | N/A, 169 | 2020/01/01-2020/06/07 | 0.91 | 0.89 |
| 10 | PMID: 33519709 | 2020 | USA | 220 | N/A, N/A | 2020/02/25-2020/06/25 | 0.33 | N/A |
| 11 | PMID: 33662839 | 2021 | USA | 593 | 11-14, N/A | 2020/01/01-2020/08/14 | N/A | 0.34 |
| 12 | PMID: 35365744 | 2022 | USA | 1356 | 6.4-8.0, N/A | 2020/03/16-2021/02/15 | 0.25 | N/A |
| 13 | PMID: 33023989 | 2020 | Italy | 90 | 8-9, N/A | 2020/02/20-2020/04/20 | N/A | 0.55 |
| 14 | PMID: 35014746 | 2022 | Italy | 31966 | 118, N/A | 2020/02/15-2020/03/15 | 0.46 | N/A |
| 15 | PMID: 32472191 | 2020 | France | 1317 | 5, 7 | 2020/03/10-2020/03/31 | 0.8 | N/A |
| 16 | PMID: 33309936 | 2020 | France | 2449 | 7,28 | 2020/03/10-2020/4/10 | 0.71 | N/A |
| 17 | PMID: 33190637 | 2020 | Spain | 1488 | N/A, N/A | 2020/03/01-2020/07/19 | 1.16 | N/A |
| 18 | doi: 10.21203/rs.3.rs-133358/v1 | 2020 | Spain | 790 | N/A, N/A | 2020/03/01-2020/05/29 | 0.98 | N/A |
| 19 | PMID: 34256824 | 2021 | Brazil | 188 | N/A, N/A | 2020/03/10-2020/11/13 | N/A | 0.33 |
| 20 | PMID: 33582839 | 2021 | South Korea | 11892 | N/A, N/A | 2020/01/01-2020/06/04 | 1.26 | N/A |
| 21 | doi: 10.1101/2020.08.20.20174169 | 2020 | UK | 190 | N/A, N/A | 2020/01/05-2020/05/27 | 0.19 | N/A |
| 22 | PMID: 34966196 | 2021 | Philippines | 278 | N/A, 30 | 2020/03/01-2020/09/30 | 0.43 | N/A |
| 23 | **Present study** | **2025** | **Japan** | **168370** | **19.4, 100** | **2021/09/01-2023/3/31** | **0.61** | **0.62** |

**Supplementary References**

1.Hayashida K, Murakami G, Matsuda S, Fushimi K. History and Profile of Diagnosis Procedure Combination (DPC): Development of a Real Data Collection System for Acute Inpatient Care in Japan. J Epidemiol 2021 January 05;31(1):1–11.

2.Clark MA, Bakhai A, Lacey MJ, Pelletier EM, Cohen DJ. Clinical and economic outcomes of percutaneous coronary interventions in the elderly: an analysis of medicare claims data. Circulation 2004 July 20;110(3):259–264.

3.Movahed MR, Ramaraj R, Hashemzadeh M, Jamal MM, Hashemzadeh M. Rate of acute ST-elevation myocardial infarction in the United States from 1988 to 2004 (from the Nationwide Inpatient Sample). Am J Cardiol 2009 July 01;104(1):5–8.

4.Ma Z, Krishnamurthy M. Is metformin use associated with low mortality in patients with type 2 diabetes mellitus hospitalized for COVID-19? a multivariable and propensity score-adjusted meta-analysis. PLoS One 2023 February 23;18(2):e0282210.
